# A comparison of the musculoskeletal assessments of the shoulder girdles of professional rugby players and professional soccer players

**DOI:** 10.1186/1758-2555-4-32

**Published:** 2012-09-10

**Authors:** Ian G Horsley, James Pearson, Ann Green, Christer Rolf

**Affiliations:** 1 English Institute of Sport, Manchester, UK; 2 Sheffield Centre for Sports Medicine, Sheffield, UK; 3 Faculty of Health and Life Sciences, Coventry, UK; 4 Karolinska Institute, Stockholm, Sweden

## Abstract

**Objective:**

To identify posture types that exist in professional rugby players, and compare them with a population of non-overhead athletes in order to identify possible relationships towards the potential for shoulder injuries.

**Design:**

Observational design Setting: Sports Medicine Clinic Participants: Convenience sample Methodology: Static assessment of posture was carried out in standing, active and passive range of glenohumeral motion, and isometric strength was carried out in accordance with previously recorded protocols.

**Interventions:**

Nil Outcome Measures: Observational classification of posture, active and passive range of glenohumeral joint range of motion, isometric strength of selected muscle groups, selected muscle flexibility and Hawkins and Neer impingement tests.

**Results:**

There was a significant difference on range of motion between the two groups (0.025–0.000), isometric middle (0.024–0.005), and lower trapezius (0.01–0.001). Conclusion: There were significant differences between strength and flexibility of muscles around the shoulder girdle between professional rugby players and a control group of professional non-overhead athletes.

## Introduction

Posture is the alignment and maintenance of body segments in specific positions [1]. Although there is not one fully accepted definition of “good posture”, it seems preferable to refer to *optimal* (or desired) and *sub-optimal* posture for the production of different activities, as it has been postulated that there is an optimal posture for any given task, and it has been suggested that certain sports may predispose participants to changes in posture [2].

The association in medicine between posture and good health has been addressed since the early part of the Twentieth Century [3,4]. The emphasis was on an “upright posture” which was described as a state of muscular balance requiring minimal muscular effort to maintain [3]. Descriptive postural recommendations were made by several authors utilizing easily identifiable bony landmarks [5,6], and more recently [7] have suggested that pain related to postural deviations is a common problem, with [8] proposing that postural alignment deviations are linked to alterations in movement patterns which will eventually lead to functional impairments.

There are numerous deviations in the observed posture within sport participants which have been proposed to be advantageous for their given sport, such as increased dominant arm external rotation in abduction for tennis players and baseball pitchers, [9], and anterior pelvic tilt for sprinters and hyperextension of the knees for swimmers [1]. Despite this, there has been little to relate static posture and how a subject will move.

The benefits of optimal posture provide both mechanical and functional benefits [10]. If body segments are held out of alignment for prolonged periods of time, the soft tissues will become shortened or lengthened [8] which will inevitably alter optimal joint range, force production and the efficiency of movement.

The nature of Rugby Union results in a relatively high risk of injury to its players, as it involves impacts, collisions at speed and vigorous body contact [11] found that the upper limb accounts for 17% of all injuries. Improper upper quarter posture, such as forward head posture [12] and an increase in thoracic kyphosis [13], is believed to be associated with glenohumeral joint pathologies [14], although not all studies agree [15,16]. Postural deviations frequently found in the cervical and thoracic spine have been suggested to affect the normal function of the glenohumeral joint [7,8,17-20].

Bloomfield [1] observed that participants from contact sports presented with abducted scapulae and rounded shoulders, with athletes assuming a tuck position when running into defenders. Previous studies have found that posture abnormalities in the upper quarter are prevalent in elite level players, but may not be as common compared to the general population [21,22], or recreational athletes [23]. For the purposes of this study, rugby was classified as an overhead sport, as previously published epidemiological studies identify the tackle situation as being responsible for between 24–58% of all injuries sustained [24]. Within the tackle position the arm of the contact shoulder is placed at approximately 90 degrees abduction.

Presently, no data currently exists on the posture of professional rugby players. This study aims to identify posture types that exist in a professional rugby squad, and compare this posture with that of a population of non-overhead athletes in order to identify relationships between posture variables and, to make inferences about potential shoulder injuries.

## Methodology

### Posture assessment

Following ethical approval from the University of Sheffield a convenience sample of 28 participants mean age 25 years (SD ± 5.0, range 19–41) from two full time professional rugby union clubs were recruited, and compared with a control group of 22 fulltime professional soccer players mean age 23.5 years (SD ± 4.8, range 18–33). The soccer players were all outfield players and thus did not utilize overhead activities regularly during their profession and did not participate in any other sport which involved overhead activities.

Inclusion criteria were; no history of shoulder or cervical or lumbar spine problems within the last 12 months, over the age of 18 years of age full time sportsmen. Any participants who were currently receiving treatment or had received treatment for upper limb or spinal problem within the last six months were excluded from the assessment. Subjects gave informed consent and were free to withdraw from the study at anytime. All assessments were carried out by the same person who had a 20 year history of working within musculo skeletal medicine.

Posture was assessed with the subjects standing comfortably and quietly in front of a plumb-line suspended from the ceiling as proposed by [25]. Subjects were exposed to the waist to allow identification of chosen landmarks; ear lobe, C7, acromion process, scapulae, thoracic spine and iliac crest. The ideal posture is detailed in Table 1. Observation in the saggital plane categorised head posture, shoulder girdle, thoracic kyphosis, lumbar spine lordosis and pelvic tilt as being operationally defined as; normal, increased or decreased. Scapulae position was defined as elevated, depressed, abducted, adducted or winging, and humeral head position was defined as normal or anterior. Fedorak [26] reported a 95% confidence interval for the mean intertester reliability for the visual assessment of cervical and lumbar lordosis utilizing a 3-level rating scale (normal, increased, or decreased) .

**Table 1:** A table to show the definition of ‘normal’ for the postural examination

**Part of Anatomy**	**Definition of ‘normal’**
Head Position	The head erect in a neutral position with an inward cervical curve.
Shoulder Position	The shoulder level slightly below the horizontal axis through T1.
Thoracic spine	A slight posterior curve of the thoracic vertebrae.
Lumbar Spine	A forward convex curve in the lumbar region.
Scapula Position	The vertebral border of the scapula is parallel to the spine and is approximately 7.5 cm from the midline of the thorax.
Humeral Head	Less than one third of the humeral head is protruding in front of the acromion.

The table was adapted from Kendall et al., (1993) [25]. Assessment.

Range of movement (Table 2), isometric strength and orthopaedic tests [29-34] were measured for both left and right glenohumeral joints in accordance with previously accepted techniques (Table 3). 

**Table 2:** Reported normal ranges of glenohumeral joint range of movement

**Range of Motion (degrees)**	**American Academy of Orthopaedic Surgeons**	**Kendall, McCreary and Provance**	**Hoppenfield**	**American Medical Association**
Flexion	0–180	0–180	0–90	0–150
Extension	0–60	0–45	0–45	0–50
Abduction	0–180	0–180	0–180	0–180
Medial Rotation	0–70	0–70	0–55	0–90
Lateral Rotation	0–90	0–90	0–45	0–90

Adapted from Norkin and White, (1995) [34].

**Table 3:** Suggested GHJ Range of Motion

**TEST**	**Method according to**
G.H.J. Range of abduction and flexion	McFarland, 2006 [27]
Glenohumeral internal rotation deficit (GIRD)	Wilk, et al. 2002 [28]
Active GH IR/ER	Magee, 1992 [29]
Passive GH IR/ER @ 90’ abduction	Sahrmann, 2002 [8]
Humeral head position	Sahrmann, 2002 [8]
Sulcus Test	Sahrmann, 2002 [8]
Hawkins impingement test	Hawkins and Kennedy, 1980 [30]
Neer’s impingement test	Neer and Welsh, 1971 [31]
*Pectoralis minor length passive	Kendal, et al. 1993 [25]
Pectoralis minor length active	Sahrmann, 2002 [8]
Pectoralis major length	Sahrmann, 2002 [8]
Upper trapezius length	Kendal, et al. 1993 [25]
Posterior Capsule tightness	Tyler, et al. 1999 [32]
Lateral and medial GHJ rotation isometric strength	Kendal, et al. 1993 [25]
Middle and lower trapezius isometric strength	Kendal, et al. 1993 [25]

* At present there are no “gold standard” reference tests for the measurement of pectoralis minor length [33].

Measurement of glenohumeral joint rotation was carried out utilizing a goniometer. Glenohumeral joint rotation was measured in a functional position- with the arm at 90° of humeral abduction utilizing a goniometer. These measurements were used to assess for Glenohumeral Internal Rotation Deficit (GIRD). GIRD is measured relative to the total motion of the glenohumeral joint [28]. Total motion is a measurement of glenohumeral internal rotation + external rotation.

Active internal rotation was also assessed by having the patient reach over the shoulder and behind the head noting what vertebral level can be reached with the thumb. Active medial rotation was assessed by asking the athlete to take his arm behind his back from below. Noting the thoracic vertebral level reached.

Table 4 – show the tests carried out – a detailed description of the test appears in the Additional file 1: Appendix.

Results show that there was a significant reduction in active range of glenohumeral joint flexion, (left p = 0.017, right = 0.025) abduction, (left p = 0.000, right = 0.000) lateral (left p = 0.001, right = 0.001) and medial rotation (left p = 0.04, right = 0.04) between the two groups. However, no significant difference was found when comparing GIRD or posterior capsular tightness. Significant differences were found between middle and lower trapezius strength between the two groups, with rugby players testing significantly stronger.

There was no significant differences between the measured isometric medial rotation strength when measured at 90 degrees abduction (left = 0.58, right = 0.33), and isometric lateral rotation strength at 90 degrees abduction, showed a significant difference on the right (0.05) and non significant difference on the left (0.293) with respect to passive range of motion. There was a significant difference between the two groups, lateral rotation (left, 0.026) medial rotation (left 0.018, right < 0.001), although there was no significant difference between right lateral rotation.

## Discussion

Changes in posture of the upper quadrant have been postulated as being responsible for shoulder problems [22] as a result of the associated muscle imbalances that accompany this. Several authors have identified alterations in the muscle force couples about the shoulder, which may be responsible for patholomechanical changes [18,22,25], although some authors have found equivocal conclusions when correlating postural differences with muscle imbalances and shoulder dysfunction [21,35,36]. Nevertheless several authors have reported that alterations in scapular resting position will alter the action of the scapulothoracic muscles, and could detrimentally affect the alignment and stabilizing forces for the humeral head within the glenoid fossa [37,38].

Loss of internal rotation has been attributed to increased tightness of the posterior structures, and has been identified in athletes with internal impingement and arthroscopically diagnosed SLAP lesions [9]. These results could be accounted for due to the conditioning training carried out by professional rugby players, as previous studies have identified an association between posterior shoulder tightness and weight training [39]. Any posterior shoulder tightness could be produced by the posterior capsule, posterior rotator cuff and/or deltoid muscle. Hung [40] reported that muscle stiffness increased in posterior deltoid, teres minor and infraspinatus when passively medially rotating the shoulder in patients with stiff shoulders, and decreased muscle stiffness when the shoulders were laterally rotated.

Increased muscle bulk around the shoulder girdle, as is evident in professional rugby players, could contribute to passive muscle tension. Since within rugby conditioning there a great emphasis placed on strengthening latissimus dorsi and pectoralis major, increased muscle bulk could be responsible for increased muscle tension towards the outer range of external rotation (as both muscles are strong medial rotators). This combined with decreased middle to inner range strength of the humeral external rotators (infraspinatus and teres minor), which could be accounted for by reduced focus to strengthening these lateral rotator muscles. Increased muscle bulk (tension) within latisimus dorsi could also account for reduced range of shoulder flexion and abduction, reduced elongation of the muscle would reduce the range of lateral humeral rotation and decrease mechanics requires at the glenohumeral joint for elevation to occur.

Within Football there is little emphasis on upper body conditioning, thus reducing the likelihood that there would be increased muscle bulk. The interaction between upper trapezius, lower trapezius and serratus anterior has been shown as being an important force couple in the production of lateral rotation of the scapula during elevation of the arm, and thus would be expected that these muscles were better developed within a population of athletes who utilized their upper limbs more with the demands of the sport; specific upper body conditioning would necessitate optimal scapular stability in order to carryout gym based training which involved overhead lifting, without complaints of shoulder pain, furthermore the demands of the game of rugby require a significant time with the arms in degrees of elevation (during the tackle, lifting at the line out and scrimmaging) which would be compromised by lack of scapulo thoracic stability. There was also a significant difference between middle and lower Trapezius strength between the two groups, with the rugby players testing significantly (P = 0.04–0.001) stronger.

This study is the first to try and describe the typical posture of a professional rugby player, and compare it to the posture of a control group of professional athletes (soccer players) who do not use their shoulders as a major component of sport-specific tasks.

In the presence of poor posture, dysfunctional muscle patterns can develop. These dysfunctions can be due to overuse, misuse, abuse or disuse [42], and the normal response to repeated muscle stress is tightness in the agonist, and in accordance with Sherrington’s Law, weakness of the antagonist due to inhibition, resulting in sub optimal movement patterns, which may predispose injury [41].

These results show that there is a significant reduction in active range of glenohumeral flexion, abduction, lateral and medial rotation between the two groups.

Interestingly, no significant differences were apparent when comparing GIRD or posterior capsule tightness, although there was a significant difference between active and passive range of internal rotation. Hence the demands for “optimal” posture for professional rugby players differ from both the presentation described by Kendall et al. (1993) and football. As both groups of subjects were asymptomatic with respect to shoulder pain at the time of assessment, one can assume that the deviations from “normal” posture and differences between sports were necessary and predisposed by the sport (rugby) itself [21,22,43].

## Conclusion

There were significant differences between strength and flexibility of muscles around the shoulder girdle between professional rugby players and a control group of professional non-overhead athletes. Further investigations will be needed to analyse if these changes are detrimental with respect to predisposing the athletes to injury, or, a postural adaptation to improve sports performance.

Further assessments are required over a larger population in order to produce normative values, and then utilised as part of a screening process, to identify possible susceptibility to injury.

Results from tests are illustrated in figures 1, 2, 3, 4, 5, 6, 7 and 8.

**Figure 1:**
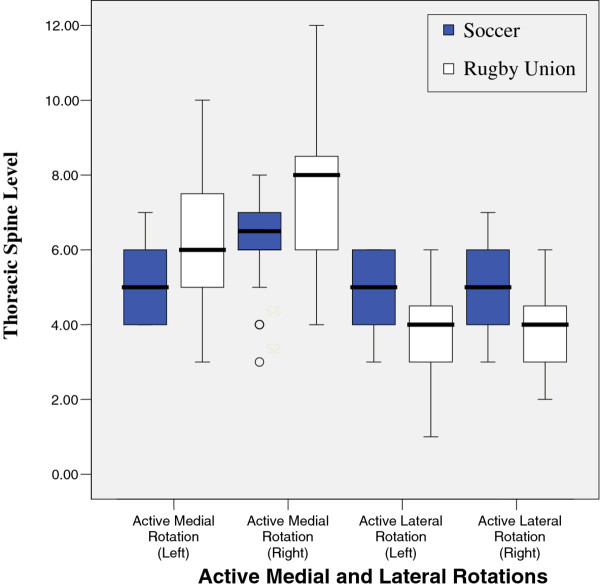
Active Medial and Lateral Rotations.

**Figure 2:**
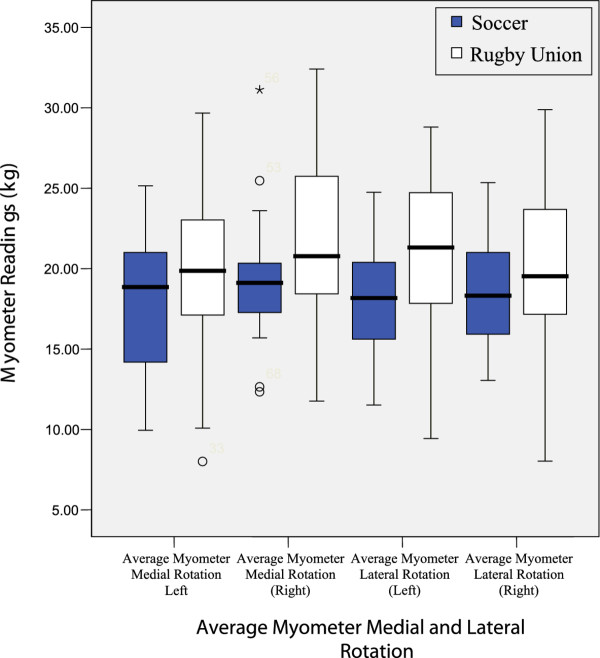
Average Myometer Medial and Lateral Rotation.

**Figure 3:**
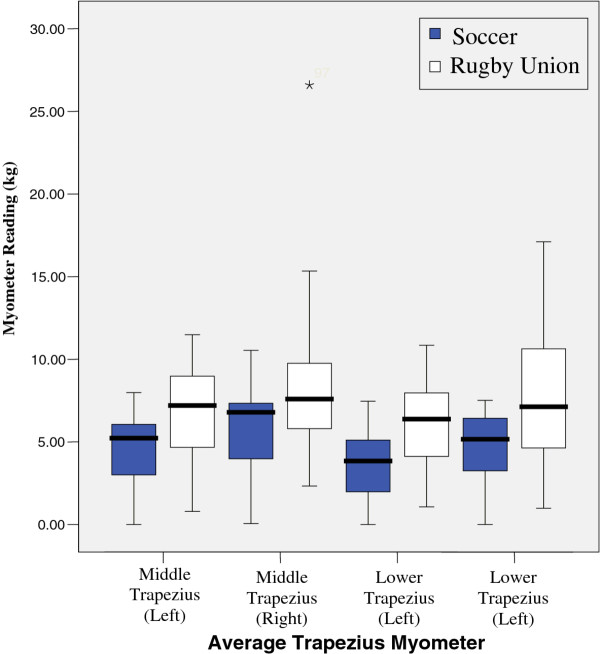
Average Trapezius Myometer.

**Figure 4:**
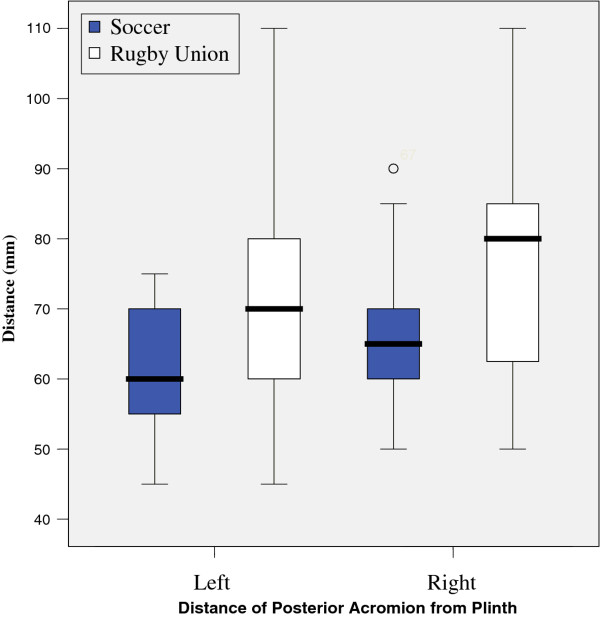
Distance of Posterior Acromion from Plinth.

**Figure 5:**
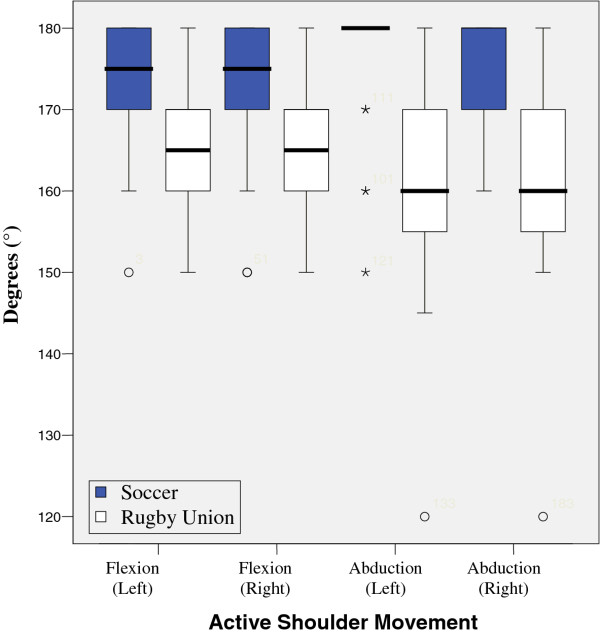
Active Shoulder Movement.

**Figure 6:**
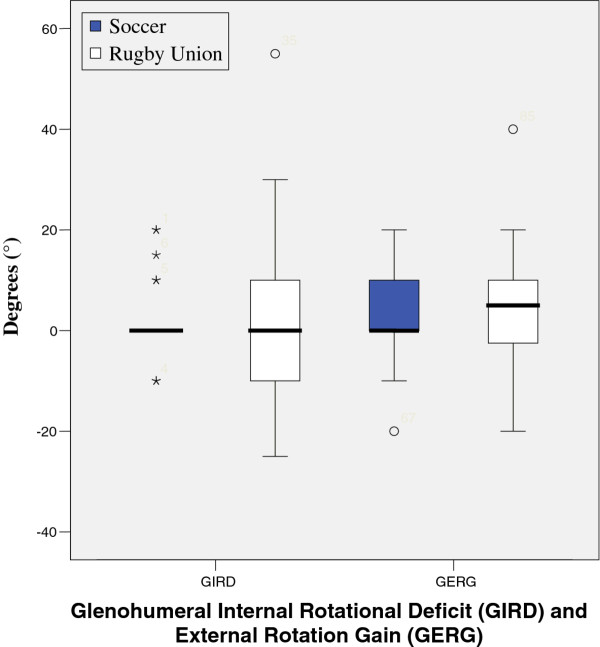
Glenohumeral Internal Rotational Deficit (GIRD) and External Rotation Gain (GERG).

**Figure 7:**
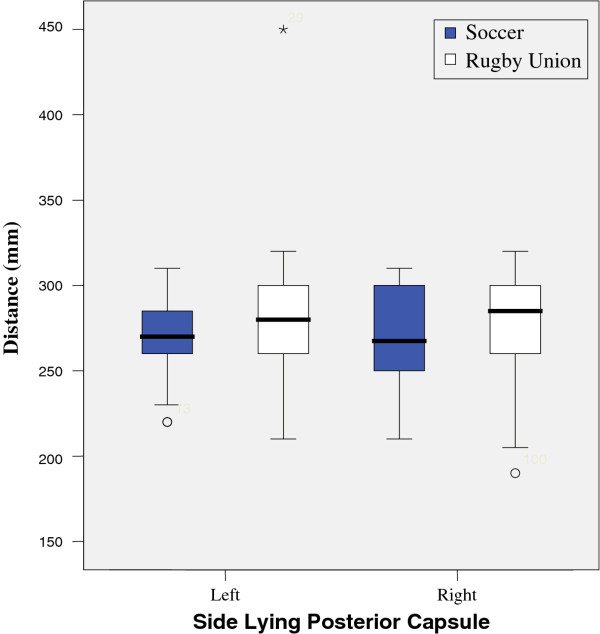
Side Lying Posterior Capsule.

**Figure 8:**
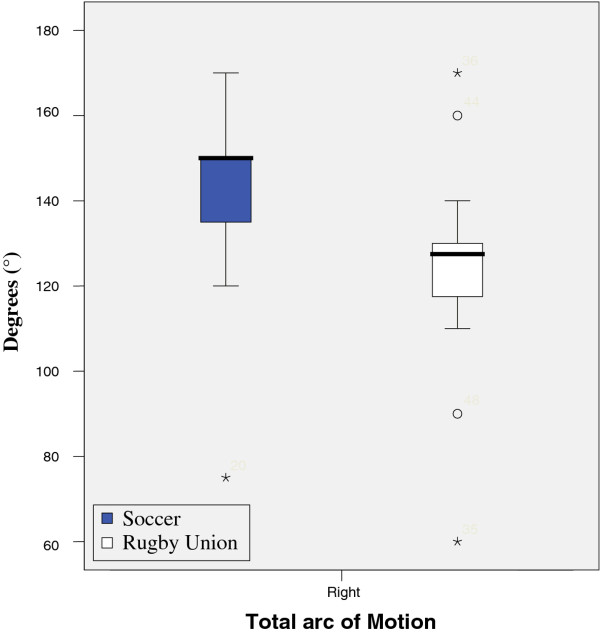
Total arc of Motion.

## Competing interests

The authors declare that they have no competing interests.

## Authors’ contributions

IGH and JP collected the data. All authors read and approved the final manuscript.

## Supplementary Material

Additional file 1 **Appendix.** Data analysis: Data were analysed using the statistical analysis software SPSS version 15 (SPSS Inc., Chicago, Il). The alpha level was set at 0.05. Results: All 50 subjects completed the study (Rugby n = 28, mean age 25.14, SD ± 5.0, range = 18–41; Control n = 22, mean age 23.95, SD ± 4.8, age range = 17–33) with no significant difference for age between groups (p = 0.24). Table (4) Anthropometric differences in shoulder girdle between rugby players and control condition. Click here for file
