# Supplementary material for: A comparison of the musculoskeletal assessments of the shoulder girdles of professional rugby players and professional soccer players
Source: Sports Med Arthrosc Rehabil Ther Technol. 2012 Sep 10;4:32. doi: 10.1186/1758-2555-4-32 (PMC3506454; doi:10.1186/1758-2555-4-32)
Supplement: Additional file 1 — Appendix. Data analysis: Data were analysed using the statistical analysis software SPSS version 15 (SPSS Inc., Chicago, Il). The alpha level was set at 0.05. Results: All 50 subjects completed the study (Rugby n = 28, mean age 25.14, SD ± 5.0, range = 18–41; Control n = 22, mean age 23.95, SD ± 4.8, age range = 17–33) with no significant difference for age between groups (p = 0.24). Table (4) Anthropometric differences in shoulder girdle between rugby players and control condition. [file 1758-2555-4-32-S1.doc]

**Data analysis**

Data were analysed using the statistical analysis software SPSS version 15 (SPSS Inc., Chicago, Il). The alpha level was set at 0.05.

**Results**

All 50 subjects completed the study (Rugby n = 28, mean age 25.14, SD ± 5.0, range = 18–41; Control n = 22, mean age 23.95, SD ± 4.8, age range = 17–33) with no significant difference for age between groups (p = 0.24).

Table (4) Anthropometric differences in shoulder girdle between rugby players and control condition

| Active GHJ | Mean (Degree) | S.D. | p-value | Significant |
| --- | --- | --- | --- | --- |
| Flexion ROM Left | 169.50 | 8.59 | 0.017 | Yes |
| Flexion ROM Right | 169.60 | 8.85 | 0.025 | Yes |
| Abduction ROM Left | 168.3 | 13.04 | 0.000 | Yes |
| Abduction ROM Right | 167.9 | 12.29 | 0.000 | Yes |
| Active Medial Rotation Left | 5.77 | 1.67 | 0.04 | Yes |
| Active Medial Rotation Right | 6.73 | 1.33 | 0.014 | Yes |
| Active Lateral Rotation Left | 4.10 | 1.25 | 0.001 | Yes |
| Active Lateral Rotation Right | 4.10 | 1.25 | 0.001 | Yes |
| GIRD | −0.90 | 13.65 | 0.618 | No |
| GERG | 3.60 | 10.40 | 0.525 | No |

| Myometer | Mean | S.D. | p-value | Significant |
| --- | --- | --- | --- | --- |
| Myometer Mid Traps Left | 6.09 | 2.52 | 0.005 | Yes |
| Myometer Mid Traps Right | 7.59 | 3.96 | 0.024 | Yes |
| Myometer Lower Traps Left | 5.15 | 2.53 | 0.01 | Yes |
| Myometer Lower Traps Right | 6.66 | 3.49 | 0.001 | Yes |
| Myometer MR 90 Degrees Abduction Left | 19.29 | 4.89 | 0.58 | No |
| Myometer MR 90 Degrees Abduction Right | 20.69 | 5.08 | 0.33 | No |
| Myometer LR 90 Degrees Abduction Left | 19.75 | 5.01 | 0.293 | No |
| Myometer LR 90 Degrees Abduction Right | 19.46 | 4.71 | 0.05 | Yes |
| Distance of Posterior Acromion from Plinth Left | 67.57 | 14.42 | 0.006 | Yes |
| Distance of Posterior Acromion from Plinth Right | 71.43 | 15.37 | 0.071 | No |
| Sd Ly Posterior Capsules |  |  |  |  |
| mm Left | 277.70 | 36.75 | 0.495 | No |
| mm Right | 272.40 | 32.04 | 0.220 | No |

| Myometer | Mean | S.D. | p-value | Significant |
| --- | --- | --- | --- | --- |
| Myometer Mid Traps Left | 6.09 | 2.52 | 0.005 | Yes |
| Myometer Mid Traps Right | 7.59 | 3.96 | 0.024 | Yes |
| Myometer Lower Traps Left | 5.15 | 2.53 | 0.01 | Yes |
| Myometer Lower Traps Right | 6.66 | 3.49 | 0.001 | Yes |
| Myometer MR 90 Degrees Abduction Left | 19.29 | 4.89 | 0.58 | No |

| Passive GHJ | Mean | S.D. | p-value | Significant |
| --- | --- | --- | --- | --- |
| Left Lateral Rotation | 84.9 | 9.34 | 0.026 | Yes |
| Right Lateral Rotation | 88.1 | 12.85 | 0.14 | No |
| Left Medial Rotation | 50.3 | 11.97 | 0.018 | Yes |
| Right Medial Rotation | 46.2 | 13.61 | <0.001 | Yes |

Range of movement of glenohumeral joint motion for abduction and flexion was measured using a standard goniometer in accordance with the methods described by [27].

## Passive lateral and medial glenohumeral rotation at 90 degrees abduction

***Tests for length of pectoralis major***

For assessment of lateral rotation, subjects lay supine on a standard treatment table with their shoulder and elbow at 90 degrees of abduction and flexion, respectively. The humerus was maintained in the cardinal plane by placing a rolled towel between the arm and the plinth, so that the acromion and the humerus were in the same plane. At the commencement of the test, the forearm was perpendicular to the floor so that the fingers were pointing towards the ceiling. This position was classed as 0 degrees of rotation. From here the examiner passively rotated the shoulder, either internally, or externally, whilst maintaining a stabilized scapula. The end of range of rotation was classed as when movement was appreciated at the acromion [8].

***Humeral head***

Both left and right humeral heads were examined to see whether they were located normally (centred on the glenoid), anterior, superior, or medially or laterally rotated. One hand was placed over the top of the shoulder so that the index finger rests on the acromion and the other hand grasps the humeral head so that the index finger is over the most anterior part and the thumb on the most posterior part. The relative position of the humeral head to the glenoid can then be assessed. There should be less than one third of the humeral head protruding beyond the acromion [8].

## Sulcus sign

Both left and right shoulders were examined for the presence of the sulcus sign. In relaxed standing, inferior traction was applied to the humerus in a controlled manor with the examiner’s inferior hand, whilst the superior hand palpated for an increase in distance of the subacromial space. A positive sulcus sign was defined as a depression between the acromion and the upper arm when inferior traction is applied to the arms [8].

*Hawkins impingement test;*

The patient’s arm was abducted to 90 degrees with the elbow flexed to 90 degrees. Pressure applied at the acromioclavicular joint whilst the shoulder is externally rotated and then internally rotated. Hawkins impingement sign is positive when pain is present, which indicates impingement [30].

The method advocated by Kendal [25] was utilized which quantified the distance from the plinth to the posterior acromion.

The patient was arranged into a supine position with their lower back flat on the table.

*Neer’s impingement test;*

The patient’s arm was flexed to 180 degrees with the elbow at full extension. Pressure was applied at the acromioclavicular joint whilst the arm was internally rotated. Neer’s impingement was recorded as positive when pain was provoked [31].

**Length assessment of sternal fibres of pectoralis major**

The arm was placed 90 degrees horizontal abduction with the elbow extended and the shoulder in lateral rotation (palm upward). If the sternal part of pectoralis major was of normal length then the arm dropped to the table level with lower back flat on the table.

However, if it was short then the extended arm did not drop down to the table level. Both left and right were classified as being normal, tight or lax [8].

**Length assessment of clavicular fibres of pectoralis major**

The arm was placed in approximately 135 degrees abduction, with the elbow extended. The shoulder was laterally rotated. If the clavicular part of pectoralis major was of normal length then the arm would be in full horizontal abduction with lateral rotation, with the arm flat on the table without trunk rotation. However, if the muscle was short then the arm did not drop down to the table level. Both left and right were classified as being normal, tight or lax [8].

**Length assessment of pectoralis minor**

Manual pressure was applied to the shoulder joint medial of the humeral head in a superior lateral direction. This should place the posterior border of the acromion in contact with the examination table. Both left and right shoulders were classified as being normal, tight or lax [8].

**Length assessment of upper fibres of trapezius**

The shoulder was fixated whilst the athlete is in supine position. The cervical spine is side flexed to the opposite ear and the range of movement is measured. Both left and right shoulders were classified as being normal, tight or lax [25].

**Posterior capsule tightness assessment**

The method chosen was that reported by Tyler [32]. The patient was placed in a side lying position on the plinth. The scapula was fixed in retraction and the upper arm was flexed to 90 degrees. The examiner then adducted the arm to capsular restriction and then measured, in centimetres, the distance from the olecranon process of the ulna to the plinth using a rule. The amount of horizontal glenohumeral adduction indicated the amount of posterior shoulder tightness; the greater the distance from the olecranon to the plinth, the tighter the posterior capsule.

**Strength tests**

***Lateral and medial rotation, middle and lower trapezius***

Lower and middle trapezius strength were measured using the positions advocated by [25]. Middle trapezius; subject prone lying with arm abducted in coronal plane to 90 degrees and externally rotated. Patient was instructed to apply maximum horizontal extension for five seconds against the resistance of the Nottingham Myometer. For lower trapezius the subject was prone with the arm flexed to 120 degrees abduction in the coronal plane, and resisted the myometer into extension for five seconds. Both tests were repeated three times on each arm. The highest value was recorded.
